# Does Culture Influence Antidepressant Response? A Preliminary Investigation of Randomized Controlled Trials of Fluoxetine

**DOI:** 10.7759/cureus.15079

**Published:** 2021-05-17

**Authors:** Ravi P Rajkumar

**Affiliations:** 1 Psychiatry, Jawaharlal Institute of Postgraduate Medical Education and Research, Pondicherry, IND

**Keywords:** power distance, hofstede’s model, fluoxetine, response rate, culture

## Abstract

Background

Contemporary models of depression view the disorder as arising from an interaction between genetic vulnerability and adverse life experiences. The nature of these experiences is strongly influenced by social-cultural factors, and there is preliminary evidence that these factors may influence the response to treatment.

Methods

In this pilot study, pooled response rates obtained from 56 randomized controlled trials of fluoxetine for major depression, conducted across 21 countries, were analyzed in relation to Hofstede’s six dimensions of culture in these countries, while controlling for methodological quality.

Results

The cultural dimensions of power distance (r = .62, p = .002), masculinity (r = .45, p = .04) and indulgence (r = -.52, p = .016) were significantly correlated with antidepressant response rates, though only the first of these remained significant after correction for multiple comparisons. On linear regression analysis, the association between power distance and antidepressant response remained significant (β = .62, p = .002).

Conclusions

These preliminary results suggest that certain cultural factors may be significantly associated with cross-national variations in antidepressant response rates during clinical trials.

## Introduction

Major depressive disorder, also known as depressive disorder or depression, is one of the leading global causes of morbidity and disability in adolescents and adults [1]. Beginning with the pioneering work of George Brown and his colleagues in the 1970s, it has been consistently observed that social factors play a significant role in influencing the onset and course of depression [2-4]. Recent research has confirmed that social factors, particularly stressful life events, can trigger the onset of depressive episodes in genetically vulnerable individuals [5,6]. According to the social signal transduction theory of depression, events or circumstances that lead to social threat cause up-regulation of immune-inflammatory pathways, which leads to symptoms of depression [7]. Similarly, the social rank hypothesis postulates that life experiences or situations which cause individuals to perceive themselves as being of a lower “rank” than others can lead to endocrine and immune dysfunction and thus to depression, particularly when such situations are chronic [8]. Both the occurrence and persistence of adverse life circumstances and individuals’ responses to them are strongly influenced by cultural factors [9, 10], which may explain the cross-cultural variations in the prevalence of depression often observed in epidemiological studies [11].

Antidepressant medications are the most widely used treatment for depression worldwide; however, response rates to these drugs vary widely between individuals, and almost half the patients receiving a given drug fail to show a satisfactory response [12]. Some of this variation is due to genetic factors affecting drug metabolism or pharmacodynamic mechanisms [13], but over the past three decades, it has also been recognized that social factors, such as the availability of social support or the occurrence of adverse life events during drug treatment, can significantly influence antidepressant response rates [14-16]. Similarly, individuals’ tendencies to seek out social support and approval have been associated with antidepressant response [17]. While some of the effect of social factors on response may be mediated by non-adherence to medication, this is not the only factor [16-18]. As these factors are themselves influenced by cultural attitudes and practices, it is possible that these cultural variables may be associated with the likelihood of response to antidepressants. However, this possibility has not been specifically examined in the literature to date. The following study represents a preliminary attempt to identify a relationship between response rates to a single antidepressant (fluoxetine) in randomized controlled trials from 21 different countries, and specific dimensions of culture in these countries.

## Materials and methods

The current study was carried out in two stages. In the first stage, the PubMed database were searched for all articles containing combinations of the terms “fluoxetine”, “depression”, “major depression”, “depressive disorder”, “major depressive disorder”, “controlled trial”, “clinical trial” and “randomized” conducted in the period 1990-2021. A total of 924 citations were retrieved. From these citations, randomized controlled trials were selected if they fulfilled the following criteria: (a) diagnosis of major depressive disorder without psychotic features, (b) randomized controlled trials comparing fluoxetine to either placebo or a comparator drug or combination of drugs, (c) acute-phase trials (4-12 weeks in duration), (d) adult participants (aged 18-65), (e) no comorbid general medical conditions, (f) response rates reported in terms of 50% reduction in scores on a standardized rating scale for depression, and (g) trials conducted in a single country. These inclusion criteria were devised to minimize the possible confounding effects of differences in antidepressant drug, dosage, duration of treatment, and variable definitions of response.

Fluoxetine was selected for the following reasons: (a) it is the first selective serotonin reuptake inhibitor (SSRI) to be approved for depression, and has been used for this indication for over 30 years; therefore, there are more controlled clinical trials available for this drug than for other SSRIs, and (b) because of its relatively low cost, it is the most frequently used SSRI in clinical trials of depression in low and middle-income countries; there are insufficient trials of other SSRIs from non-Western countries to permit a meaningful analysis [19, 20]. Further, the use of a single drug could minimize variations in response rates caused by differences in receptor binding profiles or drug-metabolizing enzyme pathways for each individual SSRI [21].

Of the 924 citations retrieved, a total of 56 randomized controlled trials of fluoxetine, conducted across 21 countries, were included in this study. Reasons for exclusion from the study included: studies conducted exclusively in patients with medical comorbidities (n = 106), studies not reporting response rates but reporting changes in other parameters, such as blood biomarker levels or physiological variables (n = 220), non-randomized, non-controlled trials (n = 109), primary psychiatric diagnosis other than major depression (n = 150), trials in children and adolescents alone (n = 73), maintenance-phase trials (n = 6), studies involving treatment-resistant subjects alone (n = 28), re-analyses or pooled analyses of other studies (n = 21), editorials, commentaries or general reviews (n = 13), multi-national studies not reporting country-wise response rates (n = 3), retracted trials (n = 2) and animal research (n = 1).

In the initial phase of the analysis, the following data on all 56 controlled trials, covering a total of 2918 subjects, was tabulated: (a) the study site, (b) the number of subjects treated, (c) the year of publication, (d) The type of trial (placebo-controlled vs. active comparator) (e) the Jadad score, indicating the methodological quality of each trial (f) response rate for each trial, defined as the percentage of fluoxetine-treated subjects showing a reduction of 50% or greater in depression severity scores on a standard rating scale [22].

Details of these trials are provided in the appendix. There was no significant correlation between the year of publication (Pearson’s r = 0.165, p = 0.225) or the sample size (r = -0.083, p = 0.543) and the response rate for each trial. There was a negative correlation between the Jadad score and the response rate, indicating that trials of lower methodological quality reported better response rates (r = -0.288, p = 0.031).

In the second stage of the analysis, country-wise weighted mean response rates were computed for each of the 21 countries, as well as mean country-wise Jadad scores. After this data was entered for each country, information on the six dimensions of culture, based on Hofstede’s six-factor model, was entered based on data obtained from the Hofstede Institute [23]. This model was derived from interviews of company employees in over eighty countries covering all continents of the world. These interviews assessed the core beliefs, values, and practices of the participants. The responses were subjected to factor analysis, which found that cultural variations at a national level could be described in terms of six orthogonal dimensions - power distance, individualism, masculinity, uncertainty avoidance, long-term orientation, and indulgence - each rated with a score ranging from 0 to 100. This work has subsequently been extended to cover 115 countries [23, 24]. Descriptions of each dimension, along with their potential relevance to antidepressant treatment, are summarized in Table 1 [25-31].

**Table 1:** Hofstede’s six-factor model of culture and its relationship to depression and antidepressant response

Factor	Description [24]	Possible relationship
Power distance	The degree to which less powerful members of a society accept and expect inequality in power distribution. Higher scores indicate a more “hierarchical” organization of society	May influence patients’ adherence to treatment [25] or expectations of response to treatment [26].
Individualism	The degree to which society privileges the individual over the group.	Depression has been associated with higher levels of cultural individualism [27].
Masculinity	A social preference for achievement, assertiveness, and competitiveness.	Higher levels of state depression [28] and lower rates of antidepressant prescription [29] have been associated with cultural masculinity.
Uncertainty avoidance	The degree to which members of a society are comfortable with uncertainty and ambiguous situations.	This cultural dimension has been associated with the prevalence of temperamental traits which may be precursors of mood disorders [30].
Long-term orientation	Indicates a preference for pragmatism, modernity, and change, as opposed to traditionalism and resistance to change	Antidepressant prescriptions may be lower in cultures with high long-term orientation [29].
Indulgence	The extent to which a society allows gratification of human drives related to pleasure or enjoyment.	Cultural indulgence is associated with higher use of antidepressants across countries [31].

The Hofstede model was selected because there is existing literature suggesting an association between some of these dimensions and the occurrence of depressive symptoms across countries [27, 28, 30], as well as with other aspects of mental health [32] and prescription patterns of antidepressants [29, 31]. Thus, it could be assumed that these factors might have an impact on the response to antidepressant drug treatment.

The correlations between each dimension of culture and the antidepressant response rate for each country were assessed using Pearson’s correlation coefficient. A post-test correction for multiple comparisons using Bonferroni’s method was applied to minimize the risk of false-positive findings. A partial correlation analysis was additionally carried out to test for the possible confounding effect of study quality, estimated using the mean Jadad score for each country. Finally, a step-wise linear regression analysis was carried out using all dimensions that were correlated with an antidepressant rate at an uncorrected significance level of less than 0.05, in order to identify potential cultural predictors of antidepressant response.

## Results

The results of the correlation analyses, both direct and corrected for the mean Jadad score are presented in Table 2. On direct analysis, three dimensions of culture were significantly associated with antidepressant response: power distance and masculinity were positively correlated with the response rate, while indulgence was negatively correlated with the response rate. Only the association between power distance and antidepressant response rate remained significant after correction for multiple comparisons (Pear

son’s r = 0.624, Pcorrected = .012, df = 19). A similar pattern was observed when the mean Jadad score was included as a covariate, except that the association between masculinity and response rate was now of marginal significance; the positive association between power distance and response rate remained significant.

**Table 2:** Direct and partial correlations between Hofstede’s dimensions of culture and antidepressant response rates * Significant at p < 0.05, uncorrected ** Significant at p < 0.05, with Bonferroni correction

Factor	Power distance	Individualism	Masculinity	Uncertainty avoidance	Long-term orientation	Indulgence
Correlation with antidepressant response rate (r)	0.624 (0.002)^**^	-0.382 (0.087)	0.451 (0.04)^*^	0.066 (0.775)	0.107 (0.645)	-0.519 (0.016)^*^
Correlation with antidepressant response rate, corrected for Jadad score (partial r)	0.610 (0.004)^**^	-0.350 (0.130)	0.440 (0.052)	0.012 (0.960)	0.058 (0.809)	-0.499 (0.025)^*^

All three variables found to be significant on bivariate correlation were entered into a stepwise multivariate linear regression analysis in order of significance. The mean Jadad score was not entered into the final model as there was no significant correlation between this variable and country-wise response rate, even on uncorrected analysis (r = -0.286, p = 0.209). In this model, only the cultural dimension of power distance (β = 0.62, p = 0.002) was associated with the response to antidepressant treatment in trials for each country.

## Discussion

The results of this study suggest that certain aspects of culture may influence the response to a short-term trial of a particular antidepressant. While these results must be viewed as preliminary, they are consistent with the existing literature which has identified a link between social factors and antidepressant response. As both social circumstances and individuals’ perceptions are influenced by culture, an association between culture and the response to psychiatric treatment is mechanistically plausible.

In this study, the cultural dimension of power distance was most significantly associated with antidepressant response. There are several mechanisms that may have mediated this association. First, there is some evidence to suggest that hierarchical cultures with a high power distance may be associated with greater peer support [33] and a clearer sense of “belonging” [34]; these factors have been associated with a better response to antidepressants in earlier analyses [14, 16]. Second, in such cultures in individuals in a position of “less power” (in this case, patients participating in a trial of antidepressants) have higher levels of trust in those considered to have “more power” (in this case, doctors or researchers) [35]. It has been suggested that this may affect patient’s expectations of being helped by treatment, leading to altered opioid peptidergic neurotransmission [36]. If this hypothesis is correct, one would also observe increased placebo response rates in these cultures. This possibility could not be tested in the current study as the majority of included trials (54 of 56) involved an active comparator. Third, cultures characterized by a large power distance are characterized by a greater tendency to obey those in a position of power [37]; this could have the effect of enhancing adherence to treatment. However, this effect is unlikely to have been of great significance in controlled clinical trials, where additional efforts are made to ensure participant adherence. Fourth, a high power distance is associated with shorter, more focused communications from doctors, characterized by clear instructions and specific information. This may have positively impacted patients’ expectations, and hence their response to treatment, in the context of a clinical trial [38]. Finally, there is evidence that cultural traits may be associated with genetic variants in specific populations through a process of co-evolution [27, 39]; if this is the case, then the observed differences may be due to variations in the frequencies of specific genetic polymorphisms, such as those related to the serotonergic system, which can themselves influence the response to antidepressants.

Though certain other cultural factors, such as masculinity and indulgence, were associated with the response to fluoxetine in bivariate analyses, these results were no longer significant after correction for multiple comparisons. However, given the potential association between these dimensions and the symptomatology and treatment of depression, analysis of a larger set of data covering a larger number of countries is necessary in order to definitively refute or confirm the tentative associations found in this study.

The results of this study are subject to certain important limitations. First, though an attempt was made to minimize confounding variables by selecting specific trials of a single drug with a uniform definition of response, it was not possible to minimize the confounding effects of other variables, either specific to the participants (such as gender, temperament, and socioeconomic circumstances) or the concerned countries (such as diet, social capital, religious or spiritual beliefs, and explanatory models of the causes and treatment of depression) [40]. Thus, it is entirely possible that the association observed in this analysis may be explained by one or more of these factors; this possibility needs to be confirmed through a prospective analysis of multi-centre antidepressant trials across a larger range of countries, allowing for the measurement of these variables. Second, while certain cultural factors may have an enhanced response in the artificial setting of a controlled clinical trial, we do not know if this effect would “translate” to a naturalistic treatment setting. In fact, there is evidence that in some community settings, a high power distance may actually serve as a barrier to effective treatment [41]. Third, the trials included in this analysis vary significantly in setting and design despite the attempts made to ensure a degree of uniformity; though the Jadad score was measured in an attempt to correct for variations in methodological quality, it is still possible that methodological factors may have affected response rates across countries. Fourth, as mentioned earlier, it is possible that the observed differences represent the effects of genetic variations across populations, in which case the observed association with culture would be epiphenomenal in nature [13, 42].

## Conclusions

In conclusion, it is possible that specific cultural factors may influence the short-term response to antidepressant medication. Given the tentative nature of this study’s findings, this possibility should be verified in prospective clinical trials that take into account both cultural and biological (pharmacogenetic and pharmacogenomic) factors, as well as the other confounding factors listed above. In such trials, both antidepressant and placebo response rates should be measured in order to identify how much of the observed variation is due to the latter. Such research would either allow this association to be confirmed with a greater degree of certainty or identify the specific confounding variables that might underlie this link.

## Appendices

Details of the 56 randomized controlled trials of fluoxetine included in the study (Table 3). 

**Table 3:** Supplementary material: details of the 56 trials included in the study *In the column "Study Type", "1" represents a placebo-controlled trial, and "2" represents an active-comparator trial.

Country	Sample Size	Year of Publication	Study Type*	Jadad	Response Rate
Australia	23	1993	1	4	57
Belgium	67	2002	2	3	50.7
Belgium	157	1996	2	2	60
Belgium	42	1995	2	4	48
Belgium	93	1994	2	3	61.3
Brazil	20	2005	2	2	56
Canada	62	1997	2	4	55.1
Canada	31	2016	2	3	29
Canada	119	1999	2	3	62
China	25	2013	2	3	60
China	34	2011	2	2	73.53
China	47	2009	2	2	77.27
China	25	2008	2	1	78.3
China	113	2008	2	4	79
Finland	105	1994	2	3	57
France	84	1992	2	2	78.9
France	46	2003	2	3	60
France	143	1999	2	3	51
Germany	114	2001	2	3	40
Germany	20	1998	2	2	65
Germany	26	1995	2	3	58
Greece	54	2000	2	4	66
India	30	2015	2	1	86.67
India	30	2013	2	4	80.33
India	30	2017	2	2	90
India	16	2013	2	3	64.7
Iran	32	2017	2	2	50
Iran	22	2015	2	5	59
Iran	16	2008	2	5	50
Iran	20	2007	2	3	85
Iran	15	2005	2	3	54
Iran	23	2011	2	4	54.54
Netherlands	45	2003	2	3	60
Pakistan	32	2013	2	3	59.4
Poland	15	2018	2	2	73
Romania	57	2008	2	5	59
Slovakia	89	2002	2	3	67
Spain	43	2009	2	1	47.18
Spain	56	2000	2	2	58.9
Sweden	54	2005	2	4	37
Turkey	28	2003	2	1	82.4
Turkey	33	2010	2	2	53
UK	68	2000	2	4	61.1
United States	120	2014	2	3	59.1
United States	12	2011	1	2	50
United States	99	2009	2	4	35
United States	28	2010	2	4	54
United States	92	2002	2	3	64.8
United States	16	2000	2	2	31
UnitedStates	35	2000	2	3	48.6
United States	54	1998	2	3	57
United States	40	1998	2	3	53
United States	68	1993	2	3	46.7
United States	29	1993	2	3	64
United States	59	1993	2	3	54.5
United States	62	1991	2	3	58

## References

[1] GBD 2019 Diseases and Injuries Collaborators (2020). Global burden of 369 diseases and injuries in 204 countries and territories, 1990-2019: a systematic analysis for the Global Burden of Disease Study 2019. Lancet.

[2] Brown GW, Harris TO, Peto J (1973). Life events and psychiatric disorders. 2. Nature of causal link. Psychol Med.

[3] Brown GW, Bifulco A, Harris T, Bridge L (1986). Life stress, chronic subclinical symptoms and vulnerability to clinical depression. J Affect Disord.

[4] Brown GW, Harris TO, Hepworth C (1994). Life events and endogenous depression. A puzzle reexamined. Arch Gen Psychiatry.

[5] Brown GW, Craig TK, Harris TO, Herbert J, Hodgson K, Tansey KE, Uher R (2014). Functional polymorphism in the brain-derived neurotrophic factor gene interacts with stressful life events but not childhood maltreatment in the etiology of depression. Depress Anxiety.

[6] Haberstick BC, Boardman JD, Wagner B (2016). Depression, stressful life events, and the impact of variation in the serotonin transporter: findings from the National Longitudinal Study of Adolescent to Adult Health (Add Health). PLoS One.

[7] Slavich GM, Irwin MR (2014). From stress to inflammation and major depressive disorder: a social signal transduction theory of depression. Psychol Bull.

[8] Wetherall K, Robb KA, O'Connor RC (2019). Social rank theory of depression: A systematic review of self-perceptions of social rank and their relationship with depressive symptoms and suicide risk. J Affect Disord.

[9] Ng RM, Bhugra D (2008). Relationship between filial piety, meta-cognitive beliefs about rumination and response style theory in depressed Chinese patients. Asian J Psychiatr.

[10] Oh H, Falbo T, Lee K (2020). Culture moderates the relationship between family obligation values and the outcomes of Korean and European American college students. J Cross Cult Psychol.

[11] Kessler RC, Bromet EJ (2013). The epidemiology of depression across cultures. Annu Rev Public Health.

[12] Bares M, Novak T, Brunovsky M, Kopecek M, Höschl C (2017). The Comparison of Effectiveness of Various Potential Predictors of Response to Treatment With SSRIs in Patients With Depressive Disorder. J Nerv Ment Dis.

[13] Kato M, Serretti A (2010). Review and meta-analysis of antidepressant pharmacogenetic findings in major depressive disorder. Mol Psychiatry.

[14] Tomaszewska W, Peselow ED, Barouche F, Fieve RR (1996). Antecedent life events, social supports and response to antidepressants in depressed patients. Acta Psychiatr Scand.

[15] Brown GW, Harris TO, Kendrick T (2010). Antidepressants, social adversity and outcome of depression in general practice. J Affect Disord.

[16] Salazar-Fraile J, Sempere-Verdú E, Pérez-Hoyos S, Tabarés-Seisdedos R, Gómez-Beneyto M (2018). Five interpersonal factors are predictive of the response to treatment of major depression with antidepressants in primary care. Front Psychiatry.

[17] Peselow ED, Robins CJ, Sanfilipo MP, Block P, Fieve RR (1992). Sociotropy and autonomy: relationship to antidepressant drug treatment response and endogenous-nonendogenous dichotomy. J Abnorm Psychol.

[18] Gentile S (2007). Extrapyramidal adverse events associated with atypical antipsychotic treatment of bipolar disorder. J Clin Psychopharmacol.

[19] Cipriani A, Brambilla P, Furukawa T (2005). Fluoxetine versus other types of pharmacotherapy for depression. Cochrane Database Syst Rev.

[20] Patel V, Chisholm D, Rabe-Hesketh S (2003). Efficacy and cost-effectiveness of drug and psychological treatments for common mental disorders in general health care in Goa, India: a randomised, controlled trial. Lancet.

[21] Carrasco JL, Sandner C (2005). Clinical effects of pharmacological variations in selective serotonin reuptake inhibitors: an overview. Int J Clin Pract.

[22] Jadad AR, Moore RA, Carroll D, Jenkinson C, Reynolds DJ, Gavaghan DJ, McQuay HJ (1996). Assessing the quality of reports of randomized clinical trials: is blinding necessary?. Control Clin Trials.

[23] (2021). The Hofstede Institute: country comparison. https://hofstede-insights.com/country-comparison.

[24] Hofstede G, Hofstede GJ, Minkov M (2010). Cultures and Organizations: Software of the Mind, Third Edition. https://books.google.co.in/books/about/Cultures_and_Organizations_Software_of_t.html?id=o4OqTgV3V00C.

[25] Shigemura J, Ogawa T, Yoshino A, Sato Y, Nomura S (2010). Predictors of antidepressant adherence: results of a Japanese Internet-based survey. Psychiatry Clin Neurosci.

[26] Benedetti F (2013). Placebo and the new physiology of the doctor-patient relationship. Physiol Rev.

[27] Chiao JY, Blizinsky KD (2010). Culture-gene coevolution of individualism-collectivism and the serotonin transporter gene. Proc Biol Sci.

[28] Arrindell WA, Steptoe A, Wardle J (2003). Higher levels of state depression in masculine than in feminine nations. Behav Res Ther.

[29] Gomez-Lumbreras A, Ferrer P, Ballarín E (2019). Study of antidepressant use in 5 European settings. Could economic, sociodemographic and cultural determinants be related to their use?. J Affect Disord.

[30] Gonda X, Vázquez GH, Akiskal KK, Akiskal HS (2011). From putative genes to temperament and culture: cultural characteristics of the distribution of dominant affective temperaments in national studies. J Affect Disord.

[31] Hoebert JM, Mantel-Teeuwisse AK, Leufkens HGM, van Dijk L (2017). Variability in market uptake of psychotropic medications in Europe reflects cultural diversity. BMC Health Serv Res.

[32] Callegari C, Ielmini M, Caselli I (2020). The 6-D model of national culture as a tool to examine cultural interpretation of migration trauma-related dissociative disorder: a case series. J Immigr Minor Health.

[33] Glazer S (2006). Social support across cultures. Int J Intercult Rel.

[34] Guo C, Tomson G, Keller C, Söderqvist F (2018). Prevalence and correlates of positive mental health in Chinese adolescents. BMC Public Health.

[35] Tyler TR, Lind EA, Huo YJ (2000). Cultural values and authority relations: the psychology of conflict resolution across cultures. Psychol Public Policy Law.

[36] Peciña M, Heffernan J, Wilson J, Zubieta JK, Dombrovski AY (2018). Prefrontal expectancy and reinforcement-driven antidepressant placebo effects. Transl Psychiatry.

[37] Merkin RS. Power distance and facework strategies (2006). J Intercult Commun Res.

[38] Meeuwesen L, van den Brink-Muinen A, Hofstede G (2009). Can dimensions of national culture predict cross-national differences in medical communication?. Patient Educ Couns.

[39] Mrazek AJ, Chiao JY, Blizinsky KD, Lun J, Gelfand MJ (2013). The role of culture-gene coevolution in morality judgment: examining the interplay between tightness-looseness and allelic variation of the serotonin transporter gene. Cult Brain.

[40] Perlman K, Benrimoh D, Israel S (2019). A systematic meta-review of predictors of antidepressant treatment outcome in major depressive disorder. J Affect Disord.

[41] Rieck AM (2014). Exploring the nature of power distance on general practitioner and community pharmacist relations in a chronic disease management context. J Interprof Care.

[42] Niitsu T, Fabbri C, Bentini F, Serretti A (2013). Pharmacogenetics in major depression: a comprehensive meta-analysis. Prog Neuropsychopharmacol Biol Psychiatry.

